# Genetic Diversity Unveiled: Cost‐Effective Methods for Grassland Species

**DOI:** 10.1111/1755-0998.70108

**Published:** 2026-02-17

**Authors:** Damian Käch, Miguel Loera‐Sánchez, Beat Reidy, Bruno Studer, Roland Kölliker

**Affiliations:** ^1^ Molecular Plant Breeding Institute of Agricultural Sciences, ETH Zurich Zurich Switzerland; ^2^ Grassland Management and Ruminant Production Systems, School of Agricultural, Forest and Food Sciences HAFL Bern University of Applied Sciences Zollikofen Switzerland; ^3^ Department of Plant Molecular Biology University of Lausanne, Quartier UNIL‐Sorge, Batiment Biophore Lausanne Switzerland

**Keywords:** genetic diversity, genotyping‐by‐sequencing, grassland, multispecies amplicon sequencing, perennial ryegrass ( *Lolium perenne* L.)

## Abstract

Permanent grasslands are predominantly composed of allogamous plant species that exhibit high levels of plant genetic diversity (PGD) within their populations. Grasslands with high PGD are more resilient to environmental stress and constitute valuable reservoirs of genetic resources for plant breeding. Therefore, monitoring PGD is the basis for detecting changes in PGD and for intervening accordingly. However, PGD monitoring is often neglected in biodiversity reports due to difficulties in taking representative samples and in using standardised and affordable indicators of PGD. Here we successfully applied two common approaches, multispecies amplicon sequencing (MSAS) and genotyping‐by‐sequencing (GBS), to assess PGD of agronomically relevant grassland species. Using MSAS, we were able to taxonomically distinguish five species ( *Dactylis glomerata* L., *Festuca pratensis* huds., *Lolium perenne* L., *Trifolium pratense* L. and *T. repens* L.) from multispecies samples and differentiate accessions within species, with fixation index (*F*_ST_) values ranging from 0.014 for *T. repens* to 0.089 for *L. perenne.* Based on an extended *L. perenne* sample set containing mixtures of two cultivars at different ratios, mixtures containing both cultivars at 50% separated from the corresponding cultivars according to this ratio using MSAS and GBS. Furthermore, GBS enabled separation of samples containing two cultivars at a 75:25 ratio from the corresponding cultivars and the 50:50‐ratio samples. These results indicate complementing applications of the two approaches in PGD monitoring. While we anticipate that MSAS with its cost‐effectiveness could be applied to large‐scale PGD monitoring, GBS with its lower detection limit could be applied to studies where cultivar composition shifts are of interest.

## 1 Introduction

Permanent grasslands are fundamental to sustainable ruminant livestock production and provide multiple ecosystem services (Schils et al. 2022). They cover 42.8% of the agricultural area of Western Europe and, in some countries, this proportion reaches over 60% (e.g., Ireland, the United Kingdom or Switzerland [Boch et al. 2020; Lüscher et al. 2019]). These grasslands are composed of economically important plant species, most of which are obligate outcrossers from the Poaceae (grass) and Fabaceae (legume) families, resulting in populations with high genetic diversity (i.e., intraspecific diversity; Last et al. 2013). This plant genetic diversity (PGD) is essential for the functioning and resilience of grassland ecosystems. It plays a crucial role in providing ecosystem services, such as reducing invertebrate herbivory (Wan et al. 2022) and stabilising biomass productivity (Meilhac et al. 2019; Prieto et al. 2015). Additionally, grasslands of high PGD constitute valuable reservoirs of genetic resources for forage plant breeding. The prospect of leveraging these benefits has led to initiatives aimed at fostering the in situ protection and management of PGD in grasslands.

Monitoring PGD in permanent grasslands is key to detect shifts in genetic diversity, intervene accordingly and to protect these valuable ecosystems. However, these changes cannot be detected through visual observations and botanical surveys and thus require molecular genetic approaches. Advances in DNA sequencing have enabled the study of PGD in many non‐model species with complex and large genomes, including grassland plant species, at continuously decreasing costs (Loera‐Sánchez et al. 2019). Sequencing‐based genetic diversity assessment is usually conducted based on single‐nucleotide polymorphism (SNP) data, which is used to estimate common genetic diversity and differentiation metrics like nucleotide diversity, heterozygosity and fixation indices (*F*_ST_). A wide range of genetic approaches is available that generate SNP data, spanning from targeted regions to entire genomes. These approaches enable the study of tens to hundreds of populations, as has been shown for some of the major plant species of permanent grasslands (Nay et al. 2023; Faville et al. 2020; Tamura et al. 2023).

Amplicon sequencing and genotyping‐by‐sequencing (GBS) are two common approaches to assess PGD in grassland species. For amplicon sequencing, a method was recently proposed as a low‐resolution (10^2^ to 10^3^ SNPs), cost‐effective approach for PGD assessment (Loera‐Sánchez et al. 2022). This multispecies amplicon sequencing (MSAS) method is based on the targeted sequencing of single‐copy, nuclear orthologous genes and ultra‐conserved‐like elements present in multiple forage grass and legume species, using MSAS primers that are transferable across multiple species within each plant family. GBS, on the other hand, uses restriction enzymes for genome complexity reduction and generates SNP data at higher resolution (10^4^ to 10^5^ SNPs) covering loci across the whole genome (Elshire et al. 2011). Therefore, MSAS and GBS differ mainly in the amount and distribution of loci that their SNP data cover, suggesting that each method could be suitable for different purposes in PGD monitoring.

Despite the availability of various molecular genetic methods and the decreasing cost of DNA sequencing, genetic diversity monitoring is often neglected in national biodiversity conservation reports worldwide (Hoban et al. 2023). This has been attributed to a lack of standardised and affordable indicators of genetic diversity in natural populations (Hoban, Campbell, et al. 2021). In the context of PGD monitoring in grassland, this could be in part due to logistical challenges to produce representative samples from large land extensions, which can harbour a great diversity of plant communities due to variations in microtopography (Deák et al. 2015). Grasslands, which commonly consist of multiple species, each represented by various accessions, pose additional questions regarding the representation of each species and accession. These complexities extend to the subsequent steps of the workflow, where appropriate genetic and bioinformatics methods have to be selected depending on the aim of PGD monitoring, whether it is detecting shifts in species or even accession composition.

In this study, we evaluate the suitability of MSAS for efficient PGD monitoring in species from permanent grasslands. Our specific objectives are to: (i) assess the genetic differentiation in pairs of accessions from five major species ( *Dactylis glomerata* L., *Festuca pratensis* huds., *Lolium perenne* L., *Trifolium pratense* L. and *T. repens* L.) using MSAS; (ii) test the suitability of MSAS to distinguish cultivars and detect cultivar shifts; and (iii) compare these results to those of GBS using an extended sample set of *L. perenne* . Based on this analysis, we discuss the prospects for long‐term, multispecies PGD monitoring in grasslands.

## 2 Materials and Methods

We used MSAS to measure genetic differentiation in pairs of accessions of five major species from permanent grasslands ( *D. glomerata* , *F. pratensis* , *L. perenne* , *T. pratense* and *T. repens* ). We further focused on six *L. perenne* cultivars to assess the detection limits and variability of genetic differentiation (i.e., fixation index, *F*_ST_) and structure metrics based on MSAS by comparing them to those produced with a genome‐wide approach (GBS).

### 2.1 Plant Material and DNA Extraction

#### 2.1.1 Seedling Pools

For the single‐accession seedling samples (SA samples), seeds of *D. glomerata* , *F. pratensis* , *L. perenne* , *T. pratense* and *T. repens* were germinated on filter paper in Petri dishes. Two accessions per species were used. We will use the label ‘A’ for the first accession of each species, and ‘B’ for the second (Table S1). For each accession, DNA extraction was performed on three biological replicates (pool of 20 seedlings per replicate). The grass seedlings were sampled at approximately 2 cm from the tip, discarding the radicle. The legume seedlings were sampled when the cotyledons emerged, discarding the radicle. The plant material was stored for a maximum of 2 weeks at −70°C in 1.5 mL tubes until processed. They were subsequently ground in 1.5 mL tubes using plastic pestles while keeping the tubes half‐submerged in liquid nitrogen. DNA was extracted from the ground material using the NucleoSpin Plant II kit (Macherey‐Nagel, Düren, Germany) resulting in 30 SA samples (five species, two accessions per species in three biological replicates).

Additionally, mixed‐species samples were prepared by pooling DNA from the SA samples. The mixed‐species seedling samples (MS samples) were prepared in triplicate for three different compositions: (1) MS‐A100, for equal DNA amounts of accessions A; (2) MS‐B100, for equal amounts of accessions B; and (3) MS‐AB50, for equal amounts of MS‐A100 and MS‐B100 (Table S2). Biological replicates of SA samples were kept separate for the preparation of MS samples resulting in nine MS samples.

#### 2.1.2 Extended *L. perenne* Sampling

We chose perennial ryegrass to conduct a wider sampling design, considering single plants and swards. For establishing the single plants, seeds of six perennial ryegrass cultivars (‘Arara’, ‘Araias’, ‘Repentinia’, ‘Artonis’, ‘Arcturus’, ‘Algira’) were germinated on filter paper in Petri dishes. All cultivars are early heading and were bred and multiplied by Agroscope (Zurich, Switzerland) and Delley seeds and plants Ltd (Delley, Switzerland; Suter et al. 2025, Suter et al. 2017). While ‘Arara’, ‘Araias’ and ‘Repentinia’ are diploid (2*n* = 2*x* = 14), ‘Artonis’, ‘Arcturus’ and ‘Algira’ are tetraploid (2*n* = 4*x* = 28) cultivars. After germination, the seedlings were transferred into pot trays (88 wells, 37 × 27 cm, compost as substrate). Pooled‐plant samples were prepared and consisted of one or two cultivars (Table 1). Single‐cultivar samples contained 30 plants represented by one leaf fragment of 5 cm per plant. For each of these cultivar compositions, three biological replicates were prepared (i.e., three replicates with 30 different plants per replicate). In addition to these pure samples, mixtures of two cultivars were prepared. These mixed samples contained a total of 60 plants (i.e., 30 plants of ‘Araias’ and 30 plants of another cultivar) at two mixing ratios. Samples with a 50:50 ratio contained leaf fragments of 5 cm with one leaf fragment per plant. Samples with a 75:25 ratio contained leaf fragments of 7.5 cm and 2.5 cm of ‘Araias’ and another cultivar, respectively, with one leaf fragment per plant.

**TABLE 1:** Cultivar compositions based on six early heading *Lolium perenne* L. cultivars bred and multiplied by Agroscope (Zurich, Switzerland) and Delley seeds and plants Ltd (Delley, Switzerland), respectively, and grown in the greenhouse and in field experiments at Eschenbach (EB) and Langenthal (LT).

Cultivar	Arara	Araias	Repentinia	Artonis	Arcturus	Algira
Ploidy	2n	2n	2n	4n	4n	4n
Parental components	22	14	7	6	9	10
Swiss cultivar since^a^	2007	2018	—	2017	2013	2013
Cultivar Composition [%]						
Ara^b^	100	—	—	—	—	—
Ari^b^	—	100	—	—	—	—
Rep^b^	—	—	100	—	—	—
Art^b^	—	—	—	100	—	—
Arc^b^	—	—	—	—	100	—
Alg^b^	—	—	—	—	—	100
Rep50^c^	—	50	50	—	—	—
EB‐Rep50^b^	—	50	50	—	—	—
LT‐Rep50^b^	—	50	50	—	—	—
Art50^c^	—	50	—	50	—	—
Alg50^c^	—	50	—	—	—	50
Rep25^c^	—	75	25	—	—	—
Art25^c^	—	75	—	25	—	—
Alg25^c^	—	75	—	—	—	25

^a^ *Source:* Suter et al. (2025).

^b^ Three biological replicates per cultivar composition.

^c^ Three technical replicates per cultivar composition.

In addition to the single plants in the greenhouse experiment, seeds of ‘Araias’ and ‘Repentinia’ were mixed at a 50:50 ratio and sown in field plots of 1.5 by 6 m at a sowing density of 500 seeds/m^2^. These cultivar compositions were grown in three replicates at two locations in Switzerland (Langenthal BE [47°; 13′3.40″ N, 7°48′15.24″ E] (LT), Eschenbach LU [47°8′16.54″ N, 8°18′16.89″ E] (EB)). After sward establishment, the plants were sampled along six parallel transects per plot. Evenly distributed along each transect, 30 plants were sampled by taking four leaf fragments of ~5 cm length per plant. For each plot, two of the six transects were randomly selected and the leaf fragments were cut to a length of 1 cm resulting in 60 plants‐pools per plot. The samples from both the greenhouse and the field experiment were stored at −80°C, freeze‐dried for 72 h and disrupted in a Qiagen TissueLyser II (Qiagen, Hilden, Germany).

DNA was extracted using the NucleoSpin II kit (Macherey‐Nagel, Düren, Germany) and its purity and concentration were assessed using a NanoDrop spectrophotometer (Thermo Fisher Scientific, Waltham, MA, USA). Based on these measurements, DNA was normalised to a concentration of 30 ng/μL. Technical replicates of the mixed samples from the greenhouse experiment were generated by dividing the normalised DNA into three samples, resulting in a total of 42 samples.

### 2.2 MSAS

Nine previously reported (Loera‐Sánchez et al. 2022) and 22 newly designed multispecies primers for grass and legume species were used for MSAS (Table 2). Primers were designed based on data from Loera‐Sánchez et al. (2022) and they target portions of single‐copy orthologous genes and ultra‐conserved‐like elements that display within‐species variability in forage grass or legume species.

**TABLE 2:** Primer pairs used for multispecies amplicon sequencing.

	Plant family	Primer pool	Amplicon name	Forward primer (5′ to 3′)	Reverse primer (5′ to 3′)
1	Fabaceae	L1	L‐1019	CAGGAGATAATCACTACYGG	TCRAARTATTTCAAYCCTRRC
2			L‐11002469	TAACTGATGCTGGACTTTCA	GCATGCCACATACAAGAAAG
3			L‐11004576^a^	AAGCTTGARGCTGAYTATGA	TGCATACCACATGACCMACT
4			L‐1262^a^	AAAGATTTGGATAGTAAAGCATGGA	TCCARCGYCCTTTYKCATCC
5			L‐1520^a^	ACACAAGCTCCTTTGTKGAA	ATACGATCATGCCACGTGTC
6		L2	L‐1031	ACACCTTCATCATCTAATGCA	ACAATGTCTCTTGCATGAGAAATG
7			L‐11002271	AGAACGTTGATNNAATMTCG	RTGACAATCAATRGMAACRGCA
8			L‐11003324	CACTTTCAAAAGTTCCCCATG	TATTCAACCARATCATTTTCAAATC
9			L‐49	TCTGGGATATAATCTCAGCT	CCTTCTTCAAYTACTATTACACTT
10		L3	L‐1348	GACAAHGTTTTCATGCTCAT	KCCCATCAGAACCATACCCA
11			L‐1390^a^	GGCACWTTCYTGAACCCTGG	GAATAACTTACTGCACTYGAGTC
12			L‐1519	TGGTYGGTGCTCTTAGGGAR	TCCATAGCAAGATCTAACTG
13	Poaceae	G1	G‐11004158^a^	GCTGGCATGACAATAAGAGC	WGTRCTCCTRAARAAGCGTGT
14			G‐11004502	ACAGAAGGGAGTAAGGCTTC	ATGGAAGTTCATGTTGGATCAA
15			G‐11005138	TTGCAAATTTCTGGTGTAGC	WGATVGAACATAACAATGGC
16			G‐1186	GATTGTVACGCCTRTTCTCA	TCCTTGTGATGAGAGGAGCAT
17			G‐1245	CTBCGTGGGGATATATCTGG	CATACCTTCCCHGGAACCAG
18			G‐1318^a^	GGGAGGAGGTGCACAAGAAG	ACAACCGRTGCCARTAACGG
19			G‐1455	TTTCCATTAATCAGGCTTCT	TCTGTATCTAACTCAAGCCC
20			G‐1542	AGTTGAAATGTATGACAAGGTCT	ATACTTCCCRGTGAGCATGC
21			G‐283	TATTATGTKCCRTGGAGGCC	ACGGCATACTTCTGGAATGA
22		G2	G‐11004715	TTTTGGTCTYGGGAATACYA	AACAAATGGTTCCTCATGAGCA
23			G‐11005008	CTGATTTCAAGCTGCCTTTC	TGGAAAGAACAAGGGATTTCC
24			G‐1288^a^	GGAAGAGATTGTTTGCAATAAAGCC	CATTATCAGTGCATCAAAGAGGA
25			G‐1347^a^	GCGTGACATTGGTCCCATGG	GATCCTBGACTCCAAGCTGTA
26			G‐1375	GAAATCTTTGAAAAGAGTGAACT	BCTGTAGGTACTTGAGAAATA
27			G‐473	KATATGRTTRCAGAAGTCYTCA	CATWGATGTCATYGAAGGRAA
28		G3	G‐1019	GCTGGAACACTTTGAGAATGA	CCAACWTCTTGCAAAAGCTT
29			G‐11005009	TACTAACCARAGCCTCRTGTAT	TCCAAATCCTGTTCCTGTAT
30			G‐1110	ATGAATGAAGAGTATTGGGA	CCATYWGGAGTRTTAAARTAAAC
31			G‐165^a^	GGAGGTGGCTACAGTCAAGT	GTGGGATGAATAGCATCTTTCATT

^a^ Primer pairs from Loera‐Sánchez et al. (2022).

Multiplex PCR was performed with the seedling samples and with the extended *L. perenne* samples. We used the Qiagen Multiplex PCR kit (Qiagen, Hilden, Germany) with 5 μL Qiagen multiplex PCR buffer, 1 μL Q‐solution, 1 μL primer mix (10 μM; three different mixes per sample; Table 2), and 15 ng of DNA in a total volume of 10 μL. In total, we used three primer mixes. The amplification conditions were: 15 min at 95°C, 30 s at 94°C, then 45 cycles of 2 min at 50°C and 1 min at 72°C. A final extension step ran for 10 min at 72°C, then reactions were held at 10°C. The resulting amplicons were pooled per sample and cleaned using AMPure beads (Beckman Coulter Life Sciences, Brea, CA, USA) using a bead‐to‐sample ratio of 0.9×.

Illumina library preparation was performed separately for the seedling samples and for the *L. perenne* extended sample set using the NEBNext Ultra II kit (New England Biolabs, Ipswich, MA, USA). Dual indexing ran for five cycles using the NEBNext Dual Index Set 1 (New England Biolabs). Samples were then cleaned with AMPure beads (0.9× proportion) and pooled equimolarly. A four‐cycle reconditioning PCR was performed for each library pool. Such a reaction consisted of 10 μL water, 12.5 μL KAPA HiFi mix (Roche, Basel, Switzerland), 1 μL i5 primer (10 μM), 1 μL i7 primer (10 μM) and 2.5 μL of the dual‐indexed library pool. The thermocycler program was: 3 min at 95°C followed by four cycles of 20 s at 98°C, 15 s at 62°C and 30 s at 72°C, followed by a final extension step of 1 min at 72°C. Library pools were spiked with 10% of phiX and sequenced on an Illumina MiSeq platform (MiSeq reagent kit v3, 2 × 300 bp; Illumina, San Diego, CA, USA) at the Genetic Diversity Center (ETH Zurich, Zurich, Switzerland).

### 2.3 GBS

GBS was conducted by LGC Genomics (Berlin, Germany) using double‐enzyme digestion (PstI‐MspI). The samples were sequenced on an Illumina NextSeq platform (2 flow cells, 1 × 75 bp; Illumina, San Diego, CA, USA). Only the extended *L. perenne* sample set was processed for GBS.

### 2.4 Data Processing and Analysis

#### 2.4.1 MSAS Variant Calling and Taxonomical Assignment

Amplicon sequencing reads were quality‐controlled with fastQC v.0.11.9 (Andrews 2010) and multiQC v1.9 (Ewels et al. 2016). Quality filtering (Q > 20), primer and adapter trimming was done using cutadapt v4.3 (Martin 2011) using the forward and reverse complement of each primer sequence. Reads from the SA samples were mapped to species‐specific reference FASTA files with the sequences of the target amplicons using minimap2 v2.17 (Li 2018). For MS samples, a FASTA file containing the sequences of the target amplicons for 16 grassland plant species was used as a reference (Loera‐Sánchez et al. 2022). Reads from the extended *L. perenne* samples were mapped to the *L. perenne* ‘Kyuss’ reference genome (Chen et al. 2024) using minimap2.

For the SA and the extended *L. perenne* samples, reads that mapped in proper pairs with MAPQ > 20 were retained using SAMtools v1.15.1 (Danecek et al. 2021; samtools view ‐Sb ‐f 2 ‐q 20). For MS samples, an additional filtering that retained only primary alignments was performed (samtools view ‐b ‐F 256 ‐q 20). For all sample types, PCR duplicates were marked using Picard v3.0.0 (Broad Institute 2019; picard MarkDuplicates) and variant calling was performed using BCFtools v1.15.1 (Danecek et al. 2021) with a maximum number of reads of 2000. Raw variant calls were filtered using BCFtools to retain bi‐allelic SNPs with a MAF = 1% and QUAL > 20.

Taxonomic assignment of MS samples was performed at the read mapping stage. This was facilitated by the sequence identifiers in the FASTA reference file, which contained the abbreviation for each species and the amplicon name. Thus, VCF files were split by assigned species.

To estimate taxonomic assignment rates, raw reads from SA samples (i.e., samples for which the species is known) were concatenated by species and then mapped to the 16 species reference FASTA. The number of reads assigned to each reference species was obtained with SAMtools (samtools idxstats). The taxonomic assignment rate was the count of reads correctly assigned to a species divided by the total read count for that species.

#### 2.4.2 GBS Variant Calling

GBS reads were demultiplexed and adapter/restriction enzyme sequences were removed by the service provider. The pre‐processed reads were then quality‐controlled and mapped as described above for amplicon sequencing. SAMtools was used to retain only primary alignments and mapped reads (samtools view ‐Sb ‐F 260) and to compute coverage (samtools coverage). Variant calling and filtering were conducted with the same parameters as amplicon sequencing. We used GNU parallel v20220522 for read processing and variant calling (Tange 2011).

#### 2.4.3 Genetic Differentiation Analysis

Allele frequencies and *F*_ST_ statistics were produced based on filtered VCF files using the R package ‘poolfstat’ v2.1.1 (Gautier et al. 2022; Hivert et al. 2018). *F*_ST_ and pairwise *F*_ST_ were calculated for single‐accession data (SA, taxonomically‐assigned MS except for MS‐AB50 and pure extended *L. perenne* ). Additionally, for each species, variants of MS samples were merged with those of SA samples, retaining only variants present in both. The resulting dataset (SAMS variants) were also used for *F*_ST_ and pairwise *F*_ST_ calculations.

For all single‐accession variant datasets, permutational multivariate analysis of variance (PERMANOVA) was performed using the function adonis2 from the R package ‘vegan’ v.2.6–2 (Oksanen et al. 2022) on euclidean distance matrices based on SNP allele frequencies, cultivar names as fixed effects and 10,000 permutations. Discriminant analysis of principal components (DAPC) for SAMS variants was performed based on their allele frequency matrices using the R package ‘adegenet’ v2.1.10 (Jombart and Ahmed 2011; Jombart 2008), retaining 4 PCA axes (i.e., considering *k*−1 for *k* = 5 a priori groups) and 5 axes for the discriminant analysis. For the pure extended *L. perenne* samples, DAPC was performed on allele frequencies. For this, the optimal numbers of PCA axes to be retained were determined by conducting cross‐validation using unscaled data and default parameters (i.e., xvalDapc(…, scale = FALSE)). The mixed samples were predicted based on that optimised DAPC using default parameters (i.e., predict.dapc(…)).

#### 2.4.4 Population Structure Analysis on the Extended *L. perenne* Samples

Population structure analysis was performed on the extended *L. perenne* samples by, first, separately calling variants in four groups of samples: (1) the Ari, Rep, Rep50, EB‐Rep50, LT‐Rep50 and Rep25 samples; (2) the Ari, Art, Art50 and Art25 samples; (3) the Ari, Alg, Alg50 and Alg25 samples; and (4) the pure samples (i.e., Ara, Ari, Rep, Art, Arc, Alg). The cultivar compositions of such samples are shown in Table 1. Each of these four groups of samples was separately analysed using ‘STRUCTURE’ v2.3 (Pritchard et al. 2000) and ‘ADMIXTURE’ v1.3 (Alexander et al. 2009). For the analysis using ‘STRUCTURE’, VCF files were converted to ‘STRUCTURE’ format using ‘PGDSpider’ v2.1.1.5. (Lischer and Excoffier 2012) and computed using the parameter settings described in Appendix S1. For the analysis using ‘ADMIXTURE’, the same VCF files were used as for ‘STRUCTURE’, were converted to BED format using ‘PLINK’ v1.9 (Purcell et al. 2007) and computed using the parameter settings described in Appendix S1.

Data processing and visualisation was conducted using the R packages ‘dplyr’ v1.0.10 (Wickham et al. 2022), ‘tidyr’ v1.2.1 (Wickham and Girlich 2022), ‘stringr’ v1.5.0 (Wickham 2022), ‘purrr’ v1.0.1 (Wickham and Henry 2023), ‘ggplot2’ v3.4.0 (Wickham 2009), ‘ggrepel’ v0.9.2 (Slowikowski 2022) and ‘ggpubr’ v0.5.0 (Kassambara 2022). Figures and calculations were performed using R v4.2.3 (R Core Team 2023) and Rstudio v2023.03.0 Build 386 ‘Cherry Blossom’ Release (RStudio Team 2020).

## 3 Results

### 3.1 Sequencing Output

The raw MSAS output for the seedling samples was 21.18 million read pairs. After read mapping and filtering, 11.93 million read pairs remained, of which 4.42 million read pairs were from SA samples and 7.52 million read pairs from MS samples.

The raw MSAS output for the extended *L. perenne* sampling was 19.03 million pair‐ended reads, which resulted in 12.45 million quality‐controlled read pairs after discarding phiX reads (3.39 million read pairs) and other undetermined reads (1.99 million read pairs), removing primer sequences and controlling for read quality and length. Samples Art25‐1 and Rep‐2 did not produce any sequencing reads and were not considered further. All amplicons were found in the ‘Kyuss’ reference genome (Table 3). 12.37 million read pairs mapped to the reference genome in proper pairs. Across all samples, average coverage per amplicon ranged from 287 ± 415 (ortho‐1375) to 133,489 ± 50,571 (ortho‐1019).

**TABLE 3:** Mapping coordinates of the amplicons in the *Lolium perenne* L. ‘Kyuss’ genome sequence.

Locus	Chromosome (chr)	Start	End
G‐283	chr1	26,882,183	26,882,675
G‐1542	chr1	142,148,139	142,148,658
G‐1455	chr2	49,666,450	49,666,893
G‐473	chr3	177,676,768	177,677,250
G‐11005138	chr4	271,713,826	271,714,378
G‐1288	chr4	271,713,865	271,714,449
G‐11005009	chr4	354,783,605	354,784,087
G‐11005008	chr4	354,784,729	354,785,194
G‐1375	chr5	28,083,648	28,084,120
G‐165	chr5	207,437,103	207,437,696
G‐11004158	chr6	25,898,407	25,898,991
G‐1186	chr6	108,414,665	108,415,028
G‐11004715	chr6	239,751,220	239,751,612
G‐1347	chr6	242,837,713	242,838,233
G‐11004502	chr6	264,461,326	264,461,599
G‐1019	chr7	53,843,084	53,843,493
G‐1245	chr7	203,254,074	203,254,613
G‐1110	chr7	224,883,315	224,883,719
G‐1318	chr7	310,725,655	310,726,210

The raw GBS output for the extended *L. perenne* sampling was 983.1 million single‐end reads. We received 897.6 million quality‐controlled reads with removed adapter sequences and restriction enzyme sites. Of these, 607.8 million reads mapped to and, on average, covered 1.7% of the ‘Kyuss’ reference genome.

### 3.2 Genetic Differentiation Between Accession Pairs in Five Major Grassland Plant Species

**FIGURE 1:**
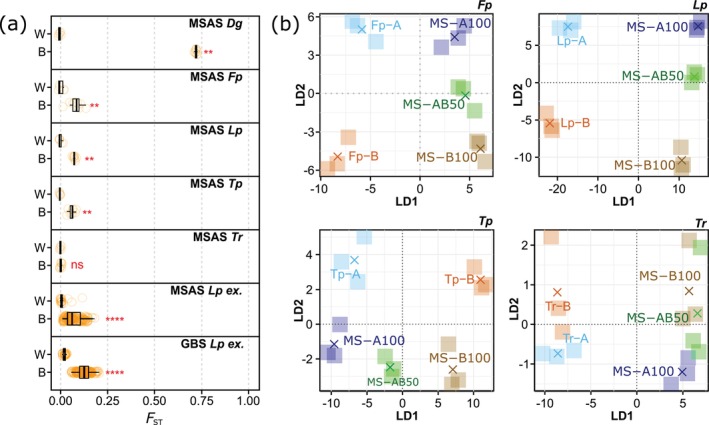
Pairwise within‐ (W) and between‐accession (B) fixation index (*F*_ST_) values based on single‐accession seedling samples of five grassland species ( *Dactylis glomerata* L. (*Dg*), *Festuca pratensis* huds. (*Fp*), *Lolium perenne* L. (*Lp*), *Trifolium pratense* L. (*Tp*), *Trifolium repens* L. (*Tr*)) and single‐cultivar samples of an extended *L. perenne* sample set (*Lp* ex.; (a)). Datasets were generated using multispecies amplicon sequencing (MSAS) or genotyping‐by‐sequencing (GBS), indicated in the panel titles. The Wilcoxon rank‐sum test significance levels are ns: *p* > 0.05, *: *p* ≤ 0.05, **: *p* ≤ 0.01, ***: *p* ≤ 0.001, ****: *p* ≤ 0.0001. Discriminant analysis of principal components separately for each species based on single‐accession seedling samples (two accessions per sample, indicated by A and B), mixtures containing accession A of all species (MS‐A100; each accession represented by 20%), mixtures containing accession B of all species (MS‐B100; each accession represented by 20%) and 50:50‐ratio mixture of MS‐A100 and MS‐B100 (MS‐AB50; (b)). Replicate means are represented by crosses and the dataset was generated using MSAS.

For SA samples, the number of variants called ranged from 86 SNPs for *T. pratense* to 802 SNPs for *D. glomerata* . Mean *F*_ST_ values ranged from 0 for *T. repens* to 0.61 for *D. glomerata* (Table 4). Between‐accessions pairwise *F*_ST_ ranged from 0.006 ± 0.005 for *T. repens* to 0.723 ± 0.006 for *D. glomerata* , while within‐accession pairwise *F*_ST_ ranged from −0.001 ± 0.009 for *T. pratense* to 0.009 ± 0.017 for *F. pratensis* . Between‐ and within‐accession pairwise *F*_ST_ values were significantly different for all species except *T. repens* (Wilcoxon rank sum test, *p* ≤ 0.05. Figure 1a). No significant differences in allele frequencies were detected by PERMANOVA (SA in Table S3).

**TABLE 4:** Single‐nucleotide polymorphism (SNP) counts, global fixation index (*F*_ST_) estimates, pairwise between‐ and within‐accession *F*_ST_ values for datasets based on single‐accession seedling samples (SA), taxonomically assigned mixed‐species seedling samples (MS), variants present in both SA and MS (SAMS; with percentage of SNPs present in SA), and single‐cultivar samples of an extended set of *Lolium perenne* L. Datasets were generated using multispecies amplicon sequencing (MSAS) or genotyping‐by‐sequencing (GBS).

Dataset	Method	Species	SNPs^a^	*F*_ST_ ^b^	*F*_ST_ (between)^c^	*F*_ST_ (within)^c^
SA	MSAS	*D. glomerata*	802	0.61	0.723 ± 0.006	0.005 ± 0.007
		*F. pratensis*	123	0.06	0.090 ± 0.035	0.009 ± 0.017
		*L. perenne*	298	0.05	0.079 ± 0.007	0.003 ± 0.011
		*T. pratense*	86	0.04	0.066 ± 0.016	0.000 ± 0.009
		*T. repens*	188	0.00	0.006 ± 0.005	0.001 ± 0.006
MS	MSAS	*F. pratensis*	61	0.022	0.043 ± 0.021	0.010 ± 0.015
		*L. perenne*	179	0.041	0.089 ± 0.022	0 ± 0.010
		*T. pratense*	141	0.044	0.080 ± 0.056	0.059 ± 0.061
		*T. repens*	178	0.008	0.014 ± 0.014	0.009 ± 0.012
SAMS	MSAS	*F. pratensis*	31 (25%)	0.068	0.098 ± 0.057	0.049 ± 0.036
		*L. perenne*	113 (38%)	0.154	0.172 ± 0.081	0.130 ± 0.106
		*T. pratense*	76 (88%)	0.051	0.091 ± 0.024	0.023 ± 0.021
		*T. repens*	149 (79%)	0.022	0.024 ± 0.018	0.019 ± 0.014
Extended *L. perenne*	MSAS	*L. perenne*	354	0.070	0.071 ± 0.042	0.017 ± 0.031
Extended *L. perenne*	GBS	*L. perenne*	43,668	0.114	0.128 ± 0.033	0.023 ± 0.008

^a^ SNP count.

^b^ Global *F*_ST_ estimate.

^c^ Mean pairwise *F*_ST_ values and standard deviation.

### 3.3 Taxonomic Assignment and Genetic Differentiation Analysis in Mixed‐Species Samples

Correct taxonomic assignment rates were 21.2%, 54.2%, 68.9%, 99.9% and 73.3% of reads for *D. glomerata* , *F. pratensis* , *L. perenne* , *T. pratense* and *T. repens* , respectively. Most frequent species incorrectly assigned were *Poa pratensis* L. (38.3% of reads) for *D. glomerata* ; *L. perenne* (40.6%) for *F. pratensis* ; *L. multiflorum* lam. (13.9%) for *L. perenne* ; and *T. pratense* (26.7%) for *T. repens* . Variant calling and filtering of the taxonomically assigned reads resulted in 61, 179, 141 and 178 SNPs in MS samples of *F. pratensis* , *L. perenne* , *T. pratense* and *T. repens* , respectively (MS in Table 4). No SNPs were obtained for taxonomically‐assigned, MS *D. glomerata* samples. Overall *F*_ST_ ranged from 0.008 ( *T. repens* ) to 0.044 ( *T. pratense* ). Between‐accessions pairwise *F*_ST_ ranged from 0.014 ± 0.014 ( *T. repens* ) to 0.089 ± 0.022 ( *F. pratensis* ). Within‐accessions pairwise *F*_ST_ ranged from 0 ± 0.010 ( *F. pratensis* ) to 0.059 ± 0.061 ( *T. pratense* ).

The differentiation pattern observed between accessions from SAMS samples was also in agreement to the pattern observed in SA samples. Variant calling and filtering resulted in 31, 113, 76 and 148 SNPs in SAMS samples *F. pratensis* , *L. perenne* , *T. pratense* and *T. repens* , respectively (SAMS in Table 4). Taxonomic assignment resulted in higher *F*_ST_ estimates for SAMS samples compared to SA samples. Overall *F*_ST_ ranged from 0.022 ( *T. repens* ) to 0.154 ( *L. perenne* ). Between‐accessions pairwise *F*_ST_ ranged from 0.0.024 ± 0.018 ( *T. repens* ) to 0.172 ± 0.081 ( *L. perenne* ). Within‐accessions pairwise *F*_ST_ ranged from 0.019 ± 0.014 ( *T. repens* ) to 0.130 ± 0.106 ( *T. pratense* ). Significant allele frequency differences were detected for *F. pratensis* and *T. pratense* by PERMANOVA, with 35.20% and 52.90%, respectively, of the total variance attributed to between‐accessions variation (SAMS in Table S3). The two accessions in each species (except for *T. repens* ) and the three types of MS samples were distinguished (Figure 1b). Furthermore, accessions were close to the corresponding MS samples and MS‐AB50 samples were located between the MS‐A100 and the MS‐B100 samples.

### 3.4 Genetic Differentiation in an Extended Sample Set of *L. perenne*

For MSAS and GBS, variant calling resulted in 354 and 43,668 SNPs, respectively. Based on pure samples, mean within‐cultivar pairwise *F*_ST_ (0.017 ± 0.031 for MSAS and 0.023 ± 0.008 for GBS) was significantly different to between‐cultivar pairwise *F*_ST_ (0.071 ± 0.042 for MSAS and 0.128 ± 0.033 for GBS) for both sequencing approaches (Figure 1a).

**FIGURE 2:**
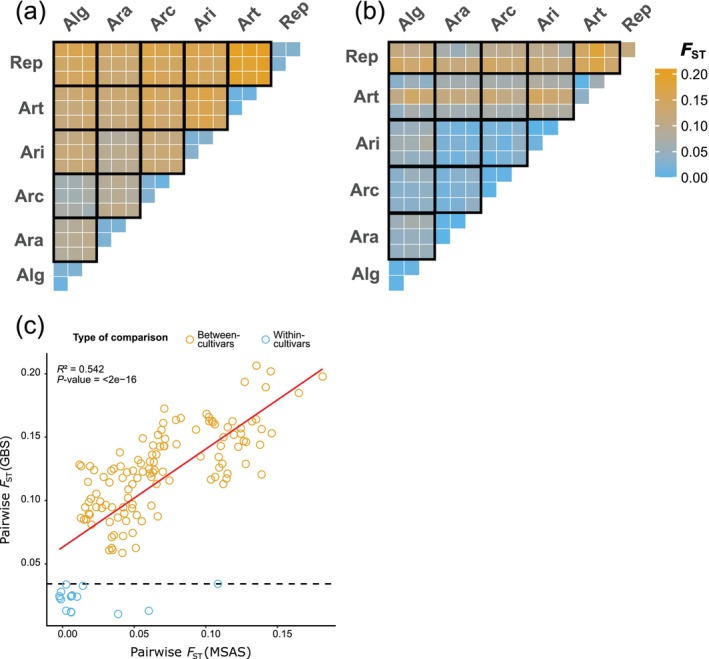
Pairwise fixation index (*F*_ST_) values between the six cultivars ‘Arara’ (Ara), ‘Araias’ (Ari), ‘Repentinia’ (Rep), ‘Artonis’ (Art), ‘Arcturus’ (Arc) and ‘Algira’ (Alg) of an extended *Lolium perenne* L. sample set analysed using genotyping‐by‐sequencing (GBS; (a)) and multispecies amplicon sequencing (MSAS; (b)). Comparison of pairwise *F*_ST_ values between GBS and MSAS (c), where beige and blue circles refer to between‐ and within‐cultivar comparisons, respectively.

The cultivars could be differentiated with both GBS and MSAS with mean *F*_ST_ values of 0.114 and 0.070, respectively (Figure 2, Table 4). Between‐cultivar *F*_ST_ values of GBS were higher than those of MSAS across all samples. However, the *F*_ST_ values of GBS significantly correlated with those of MSAS (Figure 2c). Between‐cultivar variation in GBS allele frequencies amounted to 75.34% of the total variance (PERMANOVA, *p* = 0.0001; Table S3). For MSAS allele frequencies, between‐cultivar variation was 66.86% of the total variance (PERMANOVA, *p* = 0.0001; Table S3).

For GBS, *F*_ST_ values between samples of ‘Algira’ and ‘Arcturus’ were lower compared to the other cultivars. For MSAS, the samples of ‘Artonis’ and ‘Repentinia’ had higher *F*_ST_ compared to the other cultivars. Replicates had the lowest *F*_ST_ values in both sequencing approaches. This high similarity of replicates was also reflected in high correlations of allele frequencies. Correlation coefficients between replicates were larger than 0.90 for both GBS and MSAS (Figure S1). Allele frequencies different to 0 or 1 were, however, more frequent for GBS than MSAS. Additionally, the similarity of replicates and separation of cultivars could be shown by cluster analysis (Figure S2, *K* = 6), where replicates were assigned to the same and cultivars to separate clusters. While these differentiation patterns were nearly perfect for GBS (i.e., replicates were fully assigned to the same cluster), some samples of MSAS were partially assigned to multiple clusters. Contrary to these results derived by the ‘ADMIXTURE’ analysis, cultivars could not be separated using ‘STRUCTURE’: The six cultivars were assigned to four and to one cluster for GBS and MSAS, respectively (Figure S3, *K* = 6). Nevertheless, the similarity of replicates could also be shown by similar cluster assignments using ‘STRUCTURE’.

**FIGURE 3:**
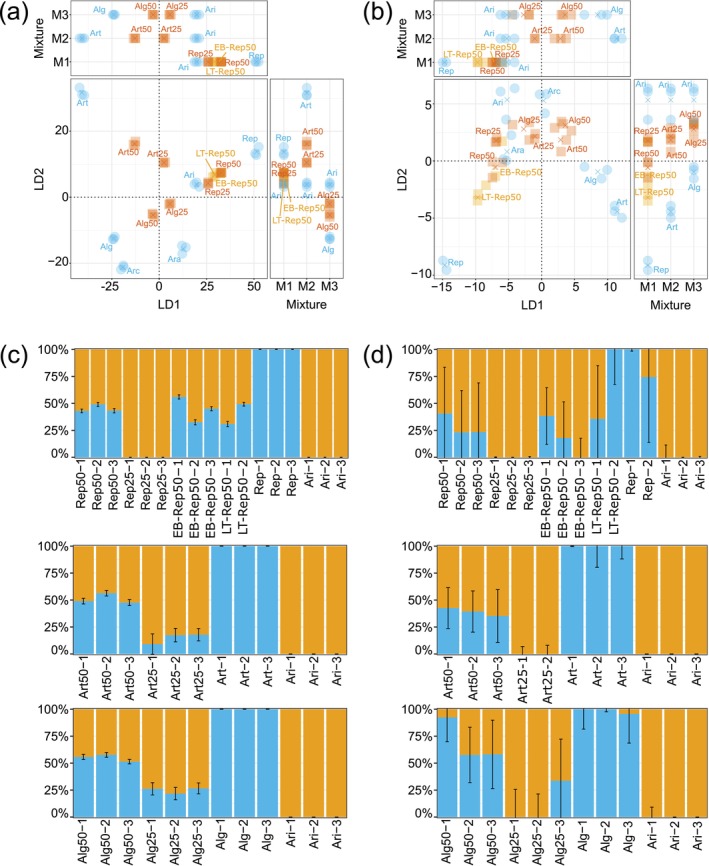
Discriminant analysis of principal components (a, b) and population structure (c, d) of an extended *Lolium perenne* L. sample set based on six cultivars: ‘Arara’ (Ara), ‘Araias’ (Ari), ‘Repentinia’ (Rep), ‘Artonis’ (Art), ‘Arcturus’ (Arc), ‘Algira’ (Alg). The sample set consists of single‐cultivar samples (blue circles), mixed samples from a greenhouse (cultivar abbreviation followed by portion of cultivar in mixture; auburn squares) and mixed samples from a field experiments (sample names with experiment location as prefix (LT for Langenthal; EB for Eschenbach); beige squares) analysed using genotyping‐by‐sequencing (a) and multispecies amplicon sequencing (b). While Ari is present in all mixtures, Rep, Art and Alg are present in M1, M2 and M3, respectively (Table 1). Replicate means are represented by crosses. Population structure analysis was based on genotyping‐by‐sequencing (c) and multispecies amplicon sequencing (d), with 95% confidence intervals. The probability of membership of each sample is indicated in beige (cluster of Ari) and blue. Sample names have the replicate number as suffix.

The cultivars could be clearly separated using a DAPC on the GBS data (Figure 3a). The DAPC was based on three PCA axes as this number resulted in the lowest mean square error during the cross‐validation procedure. Furthermore, the DAPC separated the cultivar mixtures and the corresponding cultivars according to the mixing ratios. The 50:50‐ratio samples were located in the middle of the corresponding cultivars. Additionally, the 75:25‐ratio samples, which consist of 75% of ‘Araias’ and 25% of another cultivar, were located between the corresponding 50:50‐ratio samples and ‘Araias’. Therefore, the differentiation patterns represented the genetic structure within the samples. These differentiation patterns could also be observed when separating the linear discriminants (LD). However, the differentiation pattern of LD1 was closer to the expected pattern than LD2.

The population structure assessed separately for each mixture with ‘ADMIXTURE’ was close to the expected ratios (Figure 3c). The cultivars ‘Araias’ and ‘Repentinia’ were clearly separated and assigned to two separate clusters. Mixed samples containing the two cultivars at a 50:50 ratio (i.e., Rep50) were assigned to the two clusters at a similar ratio (i.e., slightly less than 50%). This was the case for the samples from both the greenhouse and the field experiment. Mixed samples with a 75:25 ratio (i.e., Rep25) were fully, but incorrectly assigned to the same cluster ‘Araias’ belonged. The cultivars ‘Araias’ and ‘Artonis’ were clearly separated and assigned to two separate clusters. Mixed samples containing the two cultivars at a 50:50 ratio (i.e., Art50) were assigned to the two clusters at a similar ratio (i.e., approximately 50%). Mixed samples with a 75:25 ratio (i.e., Art25) were correctly assigned to the two clusters of the cultivars: while these samples were assigned to ‘Artonis’ with a probability of approximately 25%, they were accordingly assigned to ‘Araias’ with a probability of approximately 75%. The cultivars ‘Araias’ and ‘Algira’ were clearly separated and assigned to two separate clusters. Mixed samples containing the two cultivars at a 50:50 ratio (i.e., Alg50) and a 75:25 ratio (i.e., Alg25) were correctly assigned to the two clusters of the cultivars with similar separation patterns like the mixtures containing ‘Araias’ and ‘Artonis’. Results obtained with ‘STRUCTURE’ were consistent with the ‘ADMIXTURE’ analysis (Figure S4a–c).

For the MSAS data, cultivars could be clearly separated using a DAPC (Figure 3b). Based on the cross‐validation procedure, four PCA axes were retained in the DAPC. The mixed samples containing cultivars from the greenhouse experiment at a 50:50 ratio were distinguished from and located in the middle between the corresponding cultivars. However, this differentiation pattern was only visible when LD2 was included either separately or together with LD1. For LD1, the differentiation pattern was not as expected for the mixed samples containing ‘Repentinia’ or ‘Algira’ (i.e., Rep50 and Rep25). For the samples from the field experiment, samples from Langenthal and Eschenbach exclusively showed an expected differentiation pattern using LD1 and LD2 separately, respectively. The differentiation pattern of the samples containing cultivars at a 75:25 ratio was closest to the expected pattern when both LD1 and LD2 were used. When used separately, LD1 and LD2 showed expected differentiation patterns for mixed samples containing ‘Artonis’ (i.e., Art25) and ‘Repentinia’ (i.e., Rep25), respectively.

The population structure assessed separately for each mixture with ‘ADMIXTURE’ was generally not congruent with the expected ratios (Figure 3d). The cultivars ‘Araias’ and ‘Repentinia’ were separated and assigned to two separate clusters, with one exception. Mixed samples containing the two cultivars at a 50:50 ratio (i.e., Rep50) were incorrectly assigned to the two clusters of the cultivars: While these samples were assigned to ‘Repentinia’ with a probability of approximately 25% instead of 50%, they were accordingly assigned to ‘Araias’ with a probability of approximately 75% instead of 50%. Additionally, the variability between replicates was high, especially for the samples from the field experiment: While one replicate of EB‐Rep50 was fully assigned to ‘Araias’, one replicate of LT‐Rep50 was fully assigned to ‘Repentinia’. Mixed samples with a 75:25 ratio (i.e., Rep25) were fully, but incorrectly assigned to ‘Araias’. The cultivars ‘Araias’ and ‘Artonis’ were clearly separated and assigned to two separate clusters. Mixed samples containing the two cultivars at a 50:50 ratio (i.e., Art50) were slightly deviating from this ratio: While these samples were assigned to ‘Artonis’ with a probability of approximately 40%, they were accordingly assigned to ‘Araias’ with a probability of approximately 60%. Mixed samples with a 75:25 ratio (i.e., Art25) were fully, but incorrectly assigned to ‘Araias’. The cultivars ‘Araias’ and ‘Algira’ were separated and assigned to two separate clusters. Mixed samples containing the two cultivars at a 50:50 ratio (i.e., Alg50) deviated from this ratio: While two replicates were assigned to ‘Algira’ and ‘Araias’ with a probability of approximately 60% and 40%, respectively, one replicate was fully assigned to ‘Algira’. For the samples with a 75:25 ratio (i.e., Alg25), two were fully assigned to ‘Araias’ and one was assigned to ‘Algira’ and ‘Araias’ with a probability of approximately 30% and 70%, respectively. ‘STRUCTURE’ and ‘ADMIXTURE’ yielded comparable results (Figure S4d–f).

## 4 Discussion

The methods, MSAS and GBS, were successfully used in this study. The workflows used, ranging from sampling to sample preparation and bioinformatics analysis, provide insight into their applicability for PGD monitoring of grassland.

PGD monitoring in grasslands starts with collecting reliable and representative samples. This is the most crucial, but also most time‐consuming step. Increasing sampling efficiency and, therefore, decreasing time and labour would ease task allocation and would increase reliability of PGD monitoring studies as more samples could be collected (spatially and temporally). In this study, taking samples based on two parameters, number of individuals pooled in a sample and sampled leaf size, allowed highly reliable results to be obtained. For setting the pool sizes, the goal was to capture rare genotypes with a frequency of approximately 0.1. Therefore, we pooled 20 and 30 individuals per sample for the multispecies and the extended *L. perenne* to include at least one rare genotype with a frequency of 0.11 and 0.07 at a probability of 0.9, respectively (Crossa 1989; Crossa and Vencovsky 2011). Based on these pool sizes, we obtained highly reliable results confirmed by high correlations of allele frequencies between biological and technical replicates of *r* > 0.9. For the leaf size, samples were collected based solely on leaf length. Therefore, other leaf characteristics, such as leaf width, were not taken into account, although leaf width differed even within species: tetraploid *L. perenne* cultivars had up to four times broader leaves than diploid cultivars. The results, however, were still reliable, confirmed by the optimal separation pattern in mixtures of the extended *L. perenne* data set containing both diploid and tetraploid cultivars. In summary, this study contributes to more efficient PGD monitoring of grassland as samples containing only 20 to 30 individuals represented by solely leaf length provided reliable results.

Sampling for PGD monitoring is additionally time‐consuming when it comes to multispecies grassland where, commonly, each species is sampled separately (e.g., Verwimp et al. 2018). In addition to the time constraint, botanical expertise is needed to separate species. Therefore, shifting species separation to another step in the PGD monitoring workflow could open up time‐saving sampling methods, such as sampling from a pile of mown grassland species or from cow dung. In this study, we successfully shifted species separation from sampling to the computational part of the workflow. For this, we amplified MSAS target loci from different species present in a mixture and taxonomically assigned the sequences based on a reference database developed previously (Loera‐Sánchez et al. 2022) and in this study. In general, the taxonomic assignment rate for legumes was higher than that for grasses, which is consistent with what has been reported for plastome DNA barcodes (Birch et al. 2017; Loera‐Sánchez et al. 2020; Herzog and Latvis 2021). For legumes, the high taxonomic assignment rates of above 70% explain the similar genetic differentiation patterns between accessions when comparing single‐accession and multispecies samples. Even though the two legumes share similar life history (Ellison et al. 2006; Watson et al. 2000), the correct taxonomic assignment rate of *T. pratense* was higher and differed from that of *T. repens* . This could be explained by the allopolyploid nature of *T. repens* (Williams et al. 2012), leading to potentially higher divergence between the accessions used in this study and the reference MSAS sequence. For grasses, the lower taxonomic assignment rates explain the lower percentage of SNPs shared by the single‐ and multispecies seedling samples. Lower taxonomic assignment rates for grasses could be explained by their high genetic relatedness (Zhang et al. 2022) confirmed by evaluating the top incorrectly assigned species. For *F. pratensis* and *L. perenne* , these were closely related *L. perenne* and *L. multiflorum* , respectively (Cheng et al. 2016). For *D. glomerata* , however, this was *P. pratensis* which is more distantly related (Zhang et al. 2022). Additionally, the taxonomic assignment rate for *D. glomerata* was particularly lower than that of the other grasses. This could be explained by the high divergence between the candidate cultivar ‘DG1525’ (i.e., cultivar A of *D. glomerata* ) to the reference MSAS sequences of that species. In turn, this resulted in very low read mapping rates from multispecies samples to the *D. glomerata* reference sequences, so that no variant for *D. glomerata* was retained after filtering for minimum depth. Therefore, these results confirm potential difficulties in separating grassland species as the diversity within species tends to be higher than between species. Using reference genomes of germplasm more closely related to the tested cultivars, or pan‐genomes covering a broad range of diversity, could mitigate these difficulties and increase mapping rates. Nevertheless, depending on the scope of PGD monitoring, our approach of shifting species separation to the more time‐efficient computational part of PGD monitoring contributes to more efficient PGD monitoring. Therefore, our MSAS approach could be combined with even more time‐efficient sampling techniques used to analyse environmental DNA, such as sampling airborne pollen (Van Haeften et al. 2024).

After sampling for PGD monitoring, the samples are processed, sequenced and analysed. Depending on the scope of PGD monitoring, different methods can be chosen. An appropriate method should be (i) affordable, (ii) accurate and (iii) scalable (Hoban, Bruford, et al. 2021) where each method is a trade‐off between these three properties. MSAS, which is a compromise of these three properties, was investigated in this study mainly with regard to its accuracy and scalability. Previously reported MSAS‐based PGD metrics were limited to nucleotide diversity estimates in a single population. Here, we assessed the genetic differentiation among pairs of cultivars and ecotypes and observed that overall *F*_ST_ ranged from 0.04 to 0.06 (excluding *T. repens* and *D. glomerata* ). Other studies assessing genetic differentiation between cultivars and ecotypes are scarce and are mainly available for *T. pratense* and *L. perenne* . For *T. pratense* , a study focused on Nordic cultivars and wild accessions and reported an overall *F*_ST_ = 0.032 based on ~620 SNPs (Osterman et al. 2021). A *T. pratense* accession set of wider geographical origin (cultivars and European and Asian natural populations) showed an *F*_ST_ = 0.076 for ~8000 SNPs (Jones et al. 2020). For *L. perenne* , a study grouped ecotypes and cultivars into clusters based on ~500k SNPs (Blanco‐Pastor et al. 2019). The authors reported an *F*_ST_ ranging from 0.015 to 0.065 when comparing clusters containing cultivars with clusters containing ecotypes. Therefore, the genetic differentiation estimates in our study fall within the same order of magnitude as those in other studies, which supports the accuracy of our approach. Furthermore, differentiation among accessions was significantly larger than within accessions and diversity estimates were highly precise across replicates. These results indicate that the nature of SNPs targeted by MSAS (i.e., SNPs located in coding regions and ultra‐conserved‐like elements) and the resulting SNP counts obtained, which are lower compared to other studies, are useful for estimating population differentiation.

Method selection for PGD monitoring is additionally dependent on the level of accuracy desired, not only among, but also within species. Therefore, methods suitable for genetically differentiating species might not be suitable for quantitatively assessing cultivar composition within a species (i.e., proportion of each cultivar within a mixture of cultivars). The latter type of analysis could be interesting along various steps during cultivar development. It could be applied in in situ conservation of grassland which provides the base material for breeding (Peter‐Schmid et al. 2008). Beside the quality of such protected areas, detection of potential shifts in the composition within species—either due to natural fluctuations or artificially (e.g., undesired overseeding activity) – is of high interest, but poorly studied (Wambugu and Henry 2022). The method could also be applied to evaluate grassland restoration projects, where studies are scarce (Harzé et al. 2018). Beside breeding, the analysis type could be applied in variety testing. Potential variety shifts could be detected depending on various factors, such as management, which could serve as a basis for cultivars especially suited for specific managements, such as grazing. Additionally, that analysis type could be applied to assess cultivar purity (i.e., seeds only containing one cultivar) which is key for seed producers. However, these quantitative assessments of cultivar composition require methods of appropriate accuracy.

In this study, we approximated the accuracy of MSAS in a multispecies and a single‐species experiment. For the multispecies experiment, the suitability of MSAS in differentiating between accessions was evaluated. Exceptionally high genetic differentiation was detected between the candidate cultivar and the ecotype of *D. glomerata* . This high divergence might rely on the selection pressure in the candidate cultivar population during breeding, resulting in higher allele fixation compared to the ecotype (Espeland et al. 2017). Meanwhile, no genetic differentiation between the cultivar and the candidate cultivar of *T. repens* could be detected. While the divergence was expected to be lower compared to the other species where one of the two accessions was either an ecotype or a landrace, it was exceptionally lower than expected. This indicates that the genetic differentiation between both accessions falls below the detection limit of MSAS. The accuracy of MSAS was further assessed by preparing two samples where one contains accession A and the other accession B of each species. After further mixing these two mixed‐species seedling samples at a 50:50 ratio, this resulting sample could be genetically differentiated from the two samples containing only accession A or B. Additionally, the mixing ratio could be displayed in a discriminant analysis of principal components (DAPC) where the 50:50‐ratio sample was correctly located between the other two samples. The detection limit of MSAS was further evaluated by applying the method to a single‐species experiment comprising an extended set of *L. perenne* samples of cultivars mixed at different ratios. Similarly to the mixed‐species approach, the sample containing 50% of two *L. perenne* cultivars could be genetically differentiated from and were located between the two corresponding cultivars in a DAPC. However, samples containing 25% of one and 75% of the other cultivar did not segregate according to this ratio. Additionally, replicates showed high variability, which further indicates that MSAS reaches a detection limit for bi‐cultivar samples containing less than 50% of a cultivar. Therefore, these results indicate limitations of MSAS being applicable to quantitatively assess cultivar composition.

Another method for PGD monitoring is GBS, a reduced genome representation method. This method produces genetic diversity estimates of high accuracy and could be applied to quantitatively assess cultivar compositions in PGD monitoring. In contrast to MSAS, which typically targets fewer loci and thus yields fewer SNPs, GBS generates many more SNPs; accordingly, we expect higher accuracy in quantifying cultivar composition. The detection limit of GBS was evaluated and compared to that of MSAS using the same extended set of *L. perenne* samples. Similarly to MSAS, samples containing 50% of two *L. perenne* cultivars were located between the two corresponding cultivars in a DAPC. In addition to MSAS, samples containing 25% of one cultivar and 75% of ‘Araias’ segregated according to this ratio and were located between the corresponding 50:50‐sample and ‘Araias’. Additionally, variability between replicates was low confirmed by high overlaps in the DAPC. The segregation pattern and the replicate variability could be confirmed by using two other methods, ADMIXTURE and STRUCTURE, where cluster memberships represented the actual cultivar ratios. These results indicate that GBS has a lower detection limit than MSAS and could detect cultivar shifts smaller than 25% in bi‐cultivar samples. This quantitative separation of cultivar mixtures is a novel application of GBS as most other studies focused on differentiating cultivars without mixing them (e.g., Byrne et al. 2013). Verwimp et al. (2018), however, elaborated on the separation of cultivars in mixtures. The authors analysed, *inter alia*, changes in cultivar composition in mixtures containing two *L. perenne* cultivars initially sown at a 50:50 ratio. The allele frequencies of these mixtures were analysed across 4 years using a principal component analysis. As the samples came from a field experiment, the only reference on the ratio of the cultivars was the seed composition. Therefore, the authors could qualitatively track the temporal changes of cultivar mixtures, but could not further evaluate the quantitative detection limit of GBS. In the current study, the high accuracy of GBS was also confirmed by high genetic differentiation estimates between cultivars. Overall *F*_ST_ was 0.11, which was higher than that of MSAS (*F*_ST_ = 0.07) and similar to those of other studies (Blackmore et al. 2016; Blanco‐Pastor et al. 2019). These high genetic differentiation estimates and accuracy of GBS enabled reconstructing the breeding history of the cultivars. Although all cultivars contain genetic material from the same Swiss ecotype collection, their breeding history differs. ‘Algira’ and ‘Arcturus’ are closely related as they originate from the same breeding population (Grieder et al. 2015), only being selected for different heading dates, resulting in the smallest *F*_ST_ values, the closest distance on the DAPC, and the high similarity in the ADMIXTURE analysis (*K* < 6). ‘Arara’ and ‘Araias’ contain genetic material of ‘Arion’, a cultivar that is no longer on the list of recommended varieties, which resulted in smaller *F*_ST_ values and high similarity in the ADMIXTURE analysis (*K* < 5). ‘Repentinia’ and ‘Artonis’ share the fewest genetic material with the other cultivars as they originate from more distinct populations (i.a., populations from higher altitudes) resulting in the highest *F*_ST_ values, large distances on the DAPC and clear separation in the ADMIXTURE analysis (*K* > 3). This is especially true for ‘Artonis’ which is based on more distinct ecotypes collected at higher altitudes (Kempf et al. 2020) resulting in the highest separation in the ADMIXTURE analysis (*K* > 2). Therefore, DAPC and ADMIXTURE could be successfully used to reconstruct the breeding history. However, the results of the methods slightly differed when analysing the cultivar composition. While the segregation pattern was similar for most samples, that of the mixed samples containing 75% of ‘Araias’ and 25% of ‘Repentinia’ differed between the methods as the ADMIXTURE algorithm did not separate these samples from the pure ‘Araias’ samples. This is in agreement with similar studies (Tehseen et al. 2022; Deperi et al. 2018) and indicates the importance of including multiple methods to analyse genetic differentiation and approves this common practice (Miller et al. 2020).

We see great potential in MSAS and GBS being applied in PGD assessment of grassland. Both methods have strengths and limitations, making them applicable for different types of PGD analysis. MSAS could shift the labor‐intensive species separation from the sampling to the computational part of the workflow. Additionally, the genetic diversity estimates were congruent across replicates and enabled analysing population differentiation of five grassland species at high accuracy. Nevertheless, MSAS reached a detection limit when quantitatively assessing cultivar compositions of *L. perenne* . The lower detection limit of GBS, however, allowed the separation of samples containing different cultivar compositions. Although this application was exclusively tested on *L. perenne* in the current study, GBS was also used to separate grassland species in another study (Wagemaker et al. 2021). Our results indicate that MSAS and GBS could be applied to a wide range of PGD monitoring studies. While both methods are cost‐effective approaches (Hale et al. 2020; Carroll et al. 2018), MSAS is even more affordable than GBS. However, this comes at the expense of an underestimation of genetic diversity and a higher detection limit. Therefore, we anticipate that MSAS will be applied in PGD monitoring studies where resolution can be traded off for a higher sample throughput. Meanwhile, we see the application of GBS in monitoring PGD at a smaller scale, but at higher accuracy. Beside these two approaches and with a further decrease in both the sequencing cost and the cost difference between sequencing platforms (Mustafa 2024; Espinosa et al. 2024), approaches with even higher accuracy, such as whole‐genome sequencing, might find their way to PGD monitoring. However, our study highlighted the complementing nature of MSAS and GBS equipping researchers with a flexible toolkit for PGD monitoring, suitable for various study scopes and resource levels.

## Conflicts of Interest

The authors declare no conflicts of interest.

## Supporting information


**Data S1:** men70108‐sup‐0001‐DataS1.pdf.

## Data Availability

The data that support the findings of this study are openly available on Dryad at https://doi.org/10.5061/dryad.2rbnzs82f. The scripts used to process and analyse the data are openly available on GitHub at https://github.com/dkaech/gendivunveiled.
