# Supplementary material for: Genetic Diversity Unveiled: Cost‐Effective Methods for Grassland Species
Source: Mol Ecol Resour. 2026 Feb 17;26(2):e70108. doi: 10.1111/1755-0998.70108 (PMC12914157; doi:10.1111/1755-0998.70108)
Supplement: Supplementary file 1 — Data S1: men70108‐sup‐0001‐DataS1.pdf. [file MEN-26-e70108-s001.pdf]

Appendix S1

~~Supporting Information A~~ Materials and Methods

A.1 Population structure analysis on the extended *L. perenne* samples

For the analysis using 'STRUCTURE', VCFs were converted to 'STRUCTURE' format using 'PGDSpider' v2.1.1.5. (Lischer and Excoffier, 2012). In the resulting sample data files, a PopData column was added where the replicates of the same pure samples were assigned to the same population. The mixed samples were not assigned to a population (i.e., PopData value set to 0). For the groups containing both mixed and pure samples, two populations were assumed (i.e., MAXPOPS=2). The number of populations for the group containing only pure samples ranged from two to six (i.e., separate analysis for each number of populations). The ploidy, the burnin length, and the number of MCMC reps was set to diploid, 10,000, and 100,000, respectively. Additionally, admixture was assumed (i.e., NOADMIX=0), the marker type was set to co-dominant (i.e., RECESSIVEALLELES=0), and the PopData column was used (i.e., USEPOPINFO=1, except for the analysis of the pure sample group with MAXPOPS<6).

For the analysis using 'ADMIXTURE', the same VCFs were used as for 'STRUCTURE' and were converted to BED format using 'PLINK' v1.9 (Purcell et al., 2007). The optimal number of clusters (K) was assessed using a 10-fold cross-validation procedure, 2000 bootstrap replicates were used to assess the standard error. The number of clusters ranged from two to the number of cultivar compositions in the group. Additionally, the multithreaded mode was used for the analysis of the GBS data to reduce computation time.

Appendix S2

~~Supporting Information B~~ Figures and tables

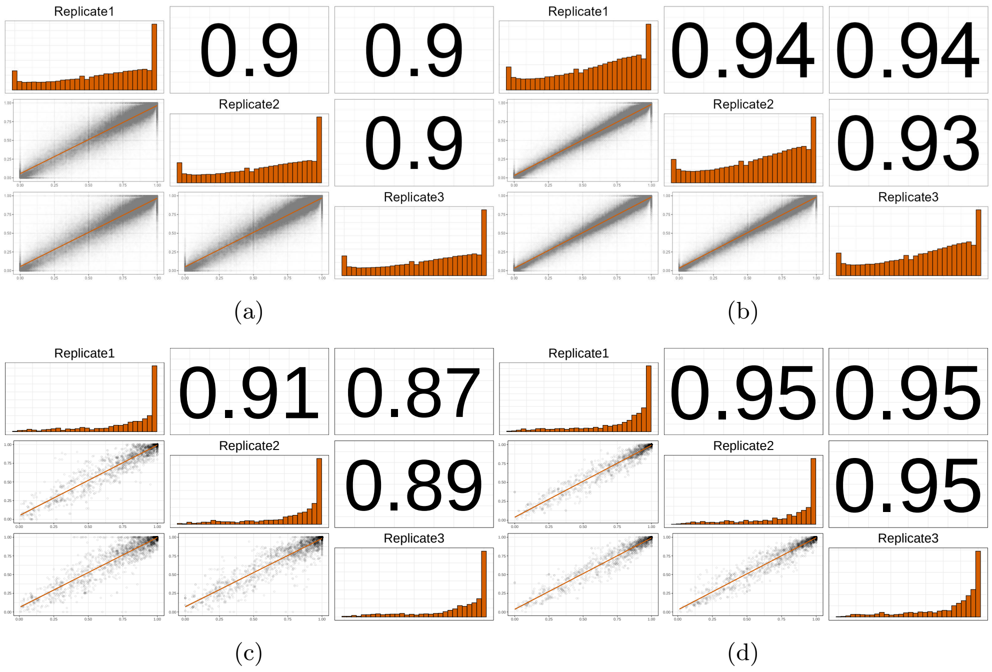

Fig. S1: Comparison of allele frequencies between replicates of the extended *Lolium perenne* L. sample set of GBS (biological (a) and technical (b) replicates) and MSAS (biological (c) and technical (d) replicates). The upper triangle contains Spearman correlation coefficients, the diagonal contains the distribution of allele frequency values, and the lower diagonal contains the frequency of each allele summarised by linear regression lines.

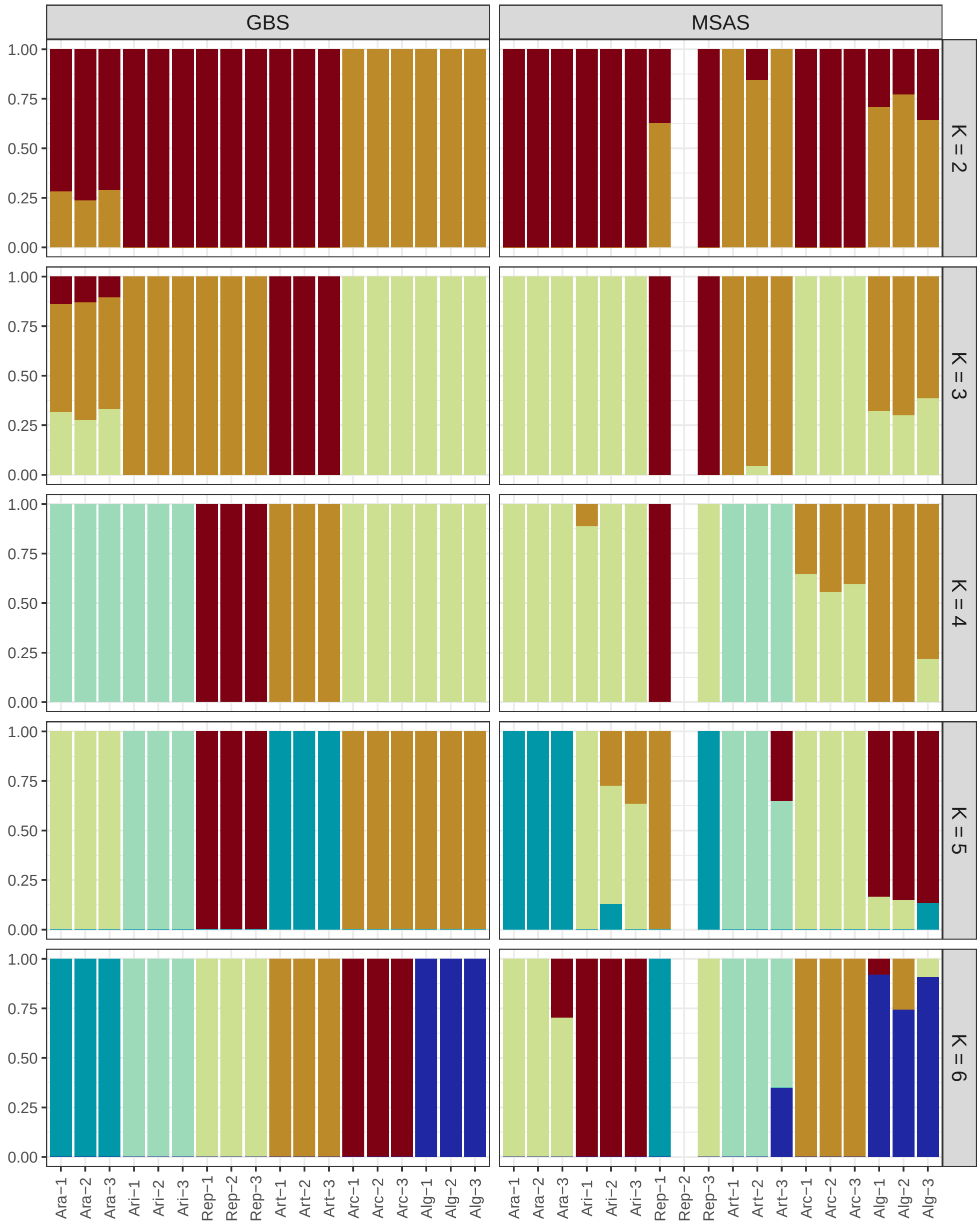

Fig. S2: Population structure (2≤ K≤ 6) based on 'ADMIXTURE' analysis of pure samples of extended *Lolium perenne* L. sample set generated with genotyping-by-sequencing (GBS) and multispecies amplicon sequencing (MSAS). The cultivar abbreviation is followed by replicate number.

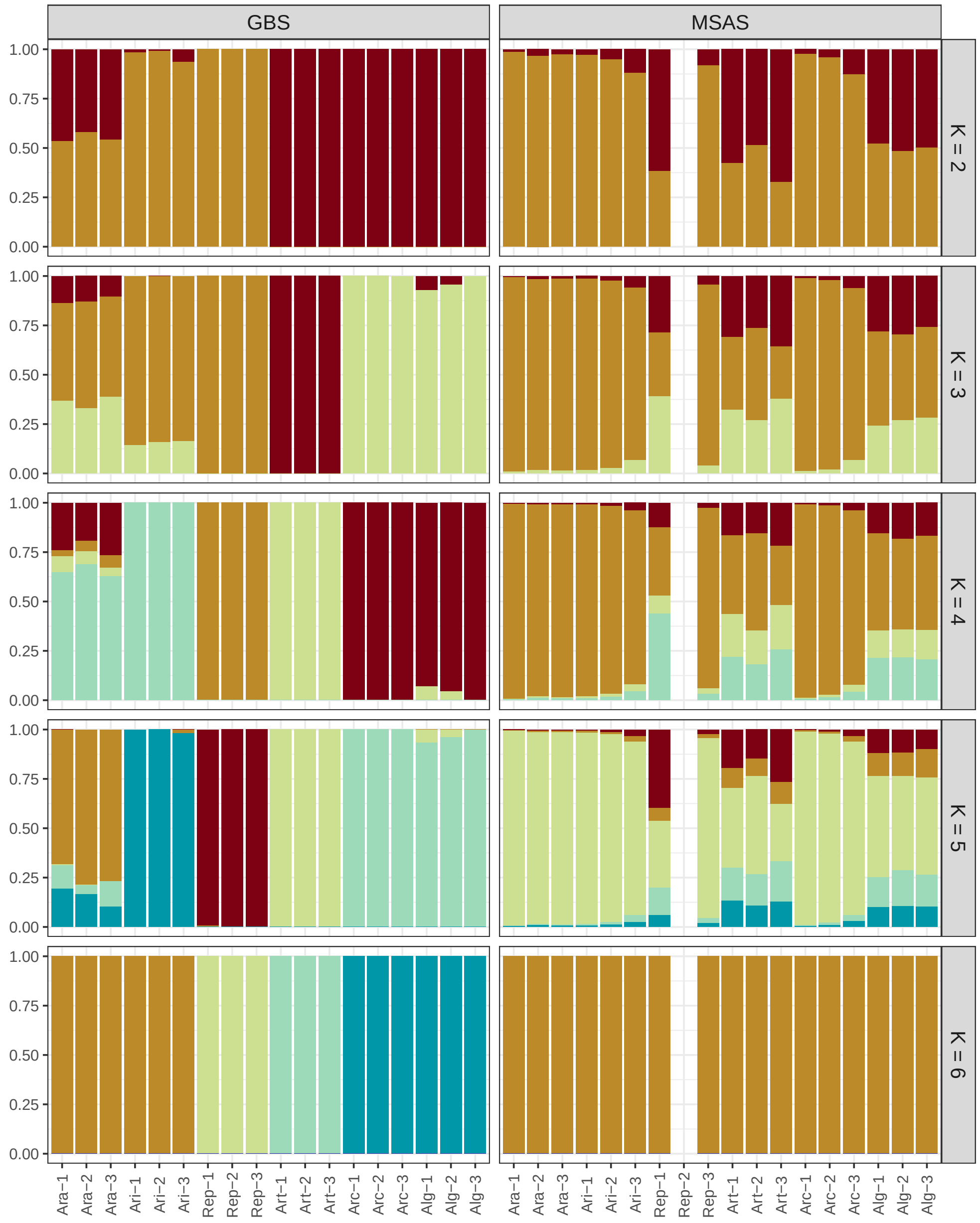

Fig. S3: Population structure (2≤ K≤ 6) based on 'STRUCTURE' analysis of pure samples of extended *Lolium perenne* L. sample set generated with genotyping-by-sequencing (GBS) and multispecies amplicon sequencing (MSAS). The cultivar abbreviation is followed by replicate number.

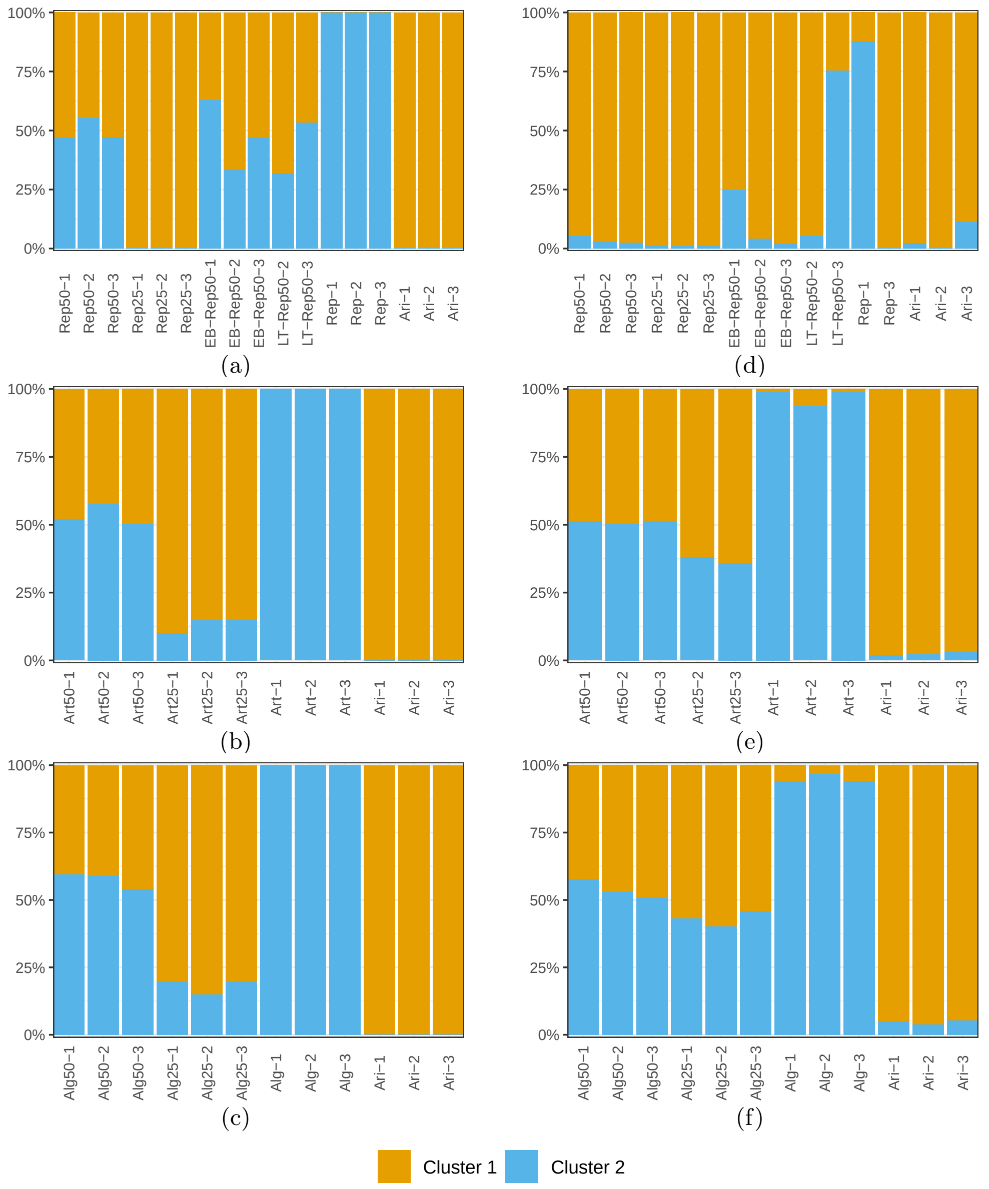

Fig. S4: Population structure based on 'STRUCTURE' analysis of the mixed samples of the extended *Lolium perenne* L. sample set generated with genotyping-by-sequencing (a-c) and multispecies amplicon sequencing (d-f). The probability of membership of each sample is indicated in beige (Cluster 1) and blue (Cluster 2). 'Araias' (Ari), which is present in all mixed samples, is mixed with 'Repentinia' (Rep; a,d), 'Artonis' (Art; b,e), and 'Algira' (Alg; c,f). For the mixed samples, the cultivar abbreviation is followed by the proportion of a cultivar in a mixture (e.g., Rep25 contains 25% Rep) and the replicate number. Samples from the field experiment correspond to Rep50 and were sown at two locations (EB and LT as sample name prefix).

Table S1: Accession compositions of the single-accession seedling samples

Sample name	Species	Accession	Description	Origin
Dg-A	*Dactylis glomerata* L.	DG1525	Candivar (6 plants; polycross)†	Agroscope, CH
Dg-B	*D. glomerata*	Bremgarten_AG_Folenweid	Ecotype	Agroscope, CH
Fp-A	*Festuca pratensis* Huds.	FP1515	Candivar (5 plants, polycross)†	Agroscope, CH
Fp-B	*F. pratensis*	Schleitheim_SH_Babental_05	Ecotype	Agroscope, CH
Lp-A	*Lolium perenne* L.	LP1715	Candivar (4 plants, polycross)†	Agroscope, CH
Lp-B	*L. perenne*	Kirchberg_SG_Tuttifrutti_99	Ecotype	Agroscope, CH
Tp-A	*Trifolium pratense* L.	'Crossway'	Cultivar	PGG Wrightson, NZ
Tp-B	*T. pratense*	Belpberg_225	Landrace	Agroscope, CH
Tr-A	*Trifolium repens* L.	'Beaumont'	Cultivar	Barenbrug, NL
Tr-B	*T. repens*	TR1205	Candivar (33 plants, open pollination)†	Agroscope, CH

† 'Candivar' indicates that the accession is currently considered a candidate variety

Table S2: Accession compositions of the mixed-species seedling samples

Sample name	Composition of DNA mixture
MS-A100	20% Dg-A<br>20% Lp-A<br>20% Fp-A<br>20% Tp-A<br>20% Tr-A
MS-B100	20% Dg-B<br>20% Lp-B<br>20% Fp-B<br>20% Tp-B<br>20% Tr-B
MS-AB50	50% MS-A100<br>50% MS-B100

Table S3: PERMANOVA results

Data	Source	Df	Sum of squares	R-squared	F	Pr(>F)	Permutations
MSAS extended *L. perenne*	Cultivar	5	37.445	0.66858	4.4381	1e-04 ***	10,000
	Residual	11	18.562	0.33142			
	Total	16	56.007	1.00000			
GBS extended *L. perenne*	Cultivar	5	14088.3	0.75335	7.3302	1e-04 ***	10,000
	Residual	12	4612.7	0.24665			
	Total	17	18701.0	1.00000			
MSAS single-species *D. glomerata*	Cultivar	1	475.45	0.98379	242.7	0.1	720
	Residual	4	7.84	0.01621			
	Total	5	483.28	1.00000			
MSAS single-species *F. pratensis*	Cultivar	1	5.3845	0.61552	6.4036	0.1	720
	Residual	4	3.3634	0.38448			
		5	8.7479	1.00000			
MSAS single-species *L. perenne*	Cultivar	1	11.7017	0.62383	6.6334	0.1	720
	Residual	4	7.0562	0.37617			
	Total	5	18.7579	1.00000			
MSAS single-species *T. pratense*	Cultivar	1	2.9371	0.63344	6.9122	0.1	720
	Residual	4	1.6996	0.36656			
	Total	5	4.6367	1.00000			
MSAS single-species *T. repens*	Cultivar	1	1.5370	0.26638	1.4524	0.1	720
	Residual	4	4.2331	0.73362			
	Total	5	5.7701	1.00000			
MSAS single- and mixed-species *F. pratensis*	Cultivar	1	2.0462	0.35199	5.4319	0.0029 **	10000
	Residual	10	3.7671	0.64801			
	Total	11	5.8133	1.00000			
MSAS single- and mixed-species *L. perenne*	Cultivar	1	8.792	0.20808	2.6275	0.082	10000
	Residual	10	33.462	0.79192			
	Total	11	42.254	1.00000			
MSAS single- and mixed-species *T. pratense*	Cultivar	1	5.6958	0.52904	11.233	0.0017 **	10000
	Residual	10	5.0705	0.47096			
	Total	11	10.7664	1.00000			
MSAS single- and mixed-species *T. repens*	Cultivar	1	1.9321	0.15175	1.789	0.1132	10000
	Residual	10	10.7999	0.84825			
	Total	11	12.7321	1.00000			

References

Lischer HEL, Excoffier L (2012) PGDSpider: An automated data conversion tool for connecting population genetics and genomics programs. Bioinformatics 28(2):298–299. https://doi.org/10.1093/bioinformatics/btr642

Purcell S, Neale B, Todd-Brown K, et al. (2007) PLINK: A tool set for whole-genome association and population-based linkage analyses. The American Journal of Human Genetics 81(3):559–575. https://doi.org/10.1086/519795
